# Management of Respiratory Distress Syndrome in Preterm Infants In Wales: A Full Audit Cycle of a Quality Improvement Project

**DOI:** 10.1038/s41598-020-60091-6

**Published:** 2020-02-26

**Authors:** Christopher Course, Mallinath Chakraborty

**Affiliations:** 1 Welsh Regional Neonatal Intensive Care Unit, University Hospital of Wales, Cardiff, UK; 2 Neonatal Registrar, HealthEducation and Improvement Wales, Cardiff, UK; 3 Cardiff University, Cardiff, UK

**Keywords:** Preterm birth, Respiratory distress syndrome

## Abstract

Respiratory Distress Syndrome (RDS) is the commonest diagnosis after premature birth. We aimed to audit clinical practices before and after introduction of a national guideline in Wales on RDS management. Anonymised, prospective data on all infants born at <34 weeks of gestation and cared for at one of the participating neonatal units in Wales were collected in two six-month time periods in 2015 and 2018. A national guideline was introduced in 2016 by the Wales Neonatal Network. Data collection included areas of antenatal management, delivery room stabilisation, invasive and non-invasive respiratory support, surfactant treatment and elements of supportive care. Univariate and multivariate methods were used to compare data between the two epochs. Comparing care before and after introduction of the national guideline, areas of significant improvement include use of targeted tidal volume ventilation, use of caffeine therapy, oxygen therapy post-surfactant and increasing early use of parenteral nutrition. Areas of poorer management included levels of positive end expiratory pressures and timing of introduction of enteral feeds. Little variation was seen between level two and three units, although gestational age was a significant independent variable for several practices, including delayed cord clamping, stabilisation with intubation, early enteral feeding and caffeine administration. A national guideline for management of RDS in Wales has significantly improved practice in several areas. However, despite a large volume of high-quality evidence and robust guidance, there remains a significant variation in some elements of best practice for RDS management. Further work should focus on education and training, especially for elements requiring cross-departmental work.

## Introduction

Respiratory pathology is one of the commonest consequences of preterm birth^1^ manifesting early as respiratory distress syndrome (RDS), a product of structurally immature lungs and pulmonary surfactant deficiency. These preterm infants often require invasive and non-invasive respiratory support, supplementary oxygen and surfactant replacement therapy. A proportion of these infants will go on to develop chronic lung disease of prematurity^2^, with abnormal respiratory function and increased respiratory morbidity persisting through childhood^3–5^ and into adult life^6^. Approximately 11% of all infants are born preterm, with this figure rising in many countries internationally^7^. Therefore, as this cohort of patients grows in number, and overall survival improves, optimal early management of these infants is likely to confer lifelong health benefits.

A wealth of research on the management of RDS has been published^8^, which was summarised by the European consensus group as best-practice guidelines^9^. This covered a broad range of care strategies related to the optimal management of RDS, including antenatal practices, early delivery room management, mechanical and non-invasive respiratory support, surfactant therapy and supportive care.

This study aimed to conduct audits on the clinical practice before and after introduction of a Welsh national guideline, describe changes in management over the past three years and highlight future directions for improving care, with broad implications for early care for preterm infants in most neonatal networks in the UK.

## Methods

Prospective, anonymised audits of the management of RDS in all preterm infants born at <34 weeks gestational age and cared for in a participating neonatal unit in Wales were undertaken. The first round of data collection was undertaken over a six-month period between September 2014 and March 2015. Following the first round of data collection, a Wales Neonatal Network Guideline on the Management of RDS, based on the European Consensus Guideline, was introduced in July 2016 and disseminated throughout all Welsh neonatal units^10^. This represented the best-practice document for the management of RDS in infants born at <34 weeks gestational age, concentrating on areas which were supported by Grade A evidence (http://www.gradeworkinggroup.org/). A second round of data collection was undertaken between March 2018 and September 2018, aiming to assess changes in practice by comparing the two cohorts.

Patient recruitment and data collection was undertaken through the Welsh Research and Education Network (WREN; www.wrenpaediatrics.com)^11^. All infants who were born at <34 weeks gestational age and were cared for in a Welsh neonatal unit were eligible for the study and all units were approached to participate.

The audit proforma was based upon the recommendations in the European Consensus Guideline on the Management of RDS in Preterm Infants 2013, and concentrated on management areas which were supported by Grade A evidence^9^. An update on the Consensus Guideline was released in 2016^12^, which did not alter the main recommendations, therefore the same proforma was used for both data collection cycles. The proforma (Supplementary Table 1) collected data on 26 items in six domains including infant demographics, antenatal management, delivery room stabilisation, surfactant management, non-invasive ventilation management, mechanical ventilation strategies and other supportive care used (Supplementary Table 1).

Data was extracted contemporaneously from written medical and nursing records, observation and prescription charts and online database records (Badgernet®, CleverMed, UK) during the infant’s management on the neonatal unit over the first two weeks of life. Data collection was continued between units for infants requiring transfer for ongoing care. Data items were coded in a standardised way and a glossary of terms was provided for clarity. A standardised, anonymised data input spreadsheet (Microsoft Excel®, Microsoft Corporation, Redmond, USA) was also created and circulated with the proforma. All data collection was anonymised once transcribed on the spreadsheet and returned to the authors via secure email.

Data was transcribed into SPSS version 25 (IBM® Corporation, New York, USA) for statistical analysis. Descriptive statistics for all variables were produced. For variables with a known eligibility denominator, univariate statistical comparisons between cohorts were made using Chi-squared and T-tests where appropriate. Unadjusted and adjusted odds ratios (OR and aOR) with 95% confidence intervals (CI) were estimated between the cohorts by logistic regression analysis using 2015 data as baseline, adjusting for level of unit of delivery (either level two or three, categorical variable) and gestational age at delivery (continuous variable). Two post-hoc subgroup analyses were performed between the two cohorts comparing infants born at below and above 28 weeks’ gestation, and for those born in level 2 or level 3 units. Statistical significance for all analyses was set at p < 0.05.

The study was designed as a quality improvement project and implemented as local audits in all participating hospitals. Local audit and governance departments of all hospitals approved the collection of routine clinical data. Individual consent was not requested from parents for the collection of routine clinical data. Anonymised data from each hospital were combined at the end of the study period for analysis; the authors had no access to any identifiable data.

## Results

Data on 225 infants was collected between September 2014 and March 2015 (2015 cohort) from four level two neonatal units and the three level three units in Wales, and on 276 infants between March and September 2018 (2018 cohort) from five level two neonatal units and three level three units in Wales. The proportion of eligible infants who participated in the data collection (included in the analysis) increased significantly in the 2018 cohort (86.5% in 2015 vs 93.9% in 2018, p < 0.01, Table 1). Demographic details for infants in both cohorts are presented in Table 1, with no significant difference being found between cohorts. The spread of gestational ages for both cohorts can be seen in the Supplementary Fig. 1. All results for both cohorts are presented in Table 2. Management flowcharts detailing all data items collected for each cohort are presented in Supplementary Figs. 2 and 3.

**Table 1:** Demographics of infants.

	2015 Cohort	2018 Cohort	p-value
Infants cared for in participating units during study period	260	294	
Infants with available data during study period	225	276	
Proportion of eligible infants included in analysis	86.5%	93.9%	<0.01
Mean Gestational Age (weeks) (std deviation)	30.3 (2.83)	30.8 (2.74)	0.14
Birthweight (g) (std deviation)	1470 (499)	1507 (481)	0.28
Born in Level 3 Unit (%)	73.8	72.1	0.4

**Table 2:** Summary of results.

	2015 Cohort (%)	2018 Cohort (%)	p-value^a^	Unadjusted OR for Cohort (95% CI)^b^	Adjusted OR for Cohort (95% CI)^c^	Adjusted OR for Level of Unit (95% CI)^d^	Adjusted OR for GA at birth (95% CI)^e^
**Antenatal Management**							
Any Antenatal Steroid Exposure	92.4	89.5	0.31	0.72 (0.39–1.36)	0.74 (0.4–1.4)	0.8 (0.39–1.64)	0.92 (0.82–1.04)
Two doses of steroids received > 24 hrs pre-delivery	70.7	70	0.30	—	—	—	—
Repeat Steroid Exposure (if eligible)	20	4.1	—	—	—	—	—
Antibiotics if PPROM?	50	43.5	—	—	—	—	—
**Delivery Room Stabilisation**							
Delayed Cord Clamping (>60 sec)	12	13	0.23	1.39 (0.81–2.38)	1.30 (0.75–2.27)	1.61 (0.85–3.06)	1.44^*^ (1.23–1.68)
Stabilised in FiO_2_ 0.21–0.3	76.4	66.3	<0.001*	0.38^*^ (0.24–0.6)	0.29^*^ (0.17–0.47)	0.79 (0.46–1.35)	1.36^*^ (1.26–1.48)
Delivered into plastic bag if GA <28 weeks	76.9	71.1	0.79	0.8 (0.15–4.24)	0.47 (0.08–2.84)	4.28 (0.59–30.91)	0.42 (0.16–1.12)
Stabilised with CPAP	50.7	51.8	0.77	1.06 (0.72–1.56)	0.78 (0.47–1.28)	1.0 (0.58–1.72)	1.85^*^ (1.65–2.07)
Intubated in delivery room	36.9	34.8	0.77	0.95 (0.64–1.39)	1.29 (0.78–2.11)	1.0 (0.58–1.73)	0.54^*^ (0.48–0.61)
**Non-Invasive Respiratory Support**							
CPAP Primary NIV used on admission	92.1	90.9	0.30	0.56 (0.19–1.67)	0.53 (0.18–1.60)	0.54 (0.15–1.97)	1.18 (0.92–1.51)
CPAP delivered through mask/binasal prongs	93	98	0.64	1.75 (0.16–19.48)	1.49 (0.13–16.99)	0.21 (0.02–2.33)	1.31 (0.56–3.06)
PEEP ≥cmH_2_O used	50.9	37.8	0.04^*^	0.54^*^ (0.33–0.89)	0.58^*^ (0.35–0.96)	1.80^*^ (1.02–3.19)	0.83^*^ (0.71–0.96)
**Mechanical Ventilation Strategies**							
TTV Used	49.1	79.1	<0.001^*^	6.97^*^ (3.38–14.36)	7.94^*^ (3.75–16.8)	1.31 (0.6–2.88)	0.88^*^ (0.77–0.99)
Caffeine used to wean MV/prevent apnoea	75.6	81.9	0.03^*^	1.84^*^ (1.07–3.17)	2.49^*^ (1.4–4.6)	0.89 (0.48–1.69)	0.56^*^ (0.45–0.69)
Course of Dexamethasone used if MV at 1–2 weeks	17.5	35.3	—	—	—	—	—
**Surfactant Therapy**							
If intubated received surfactant	100	97.9	0.19	—	—	—	—
Received natural surfactant	100	100	—	—	—	—	—
Received 200 mg/kg dose	49.5	45.7	0.26	1.4 (0.78–2.51)	1.38 (0.77–2.49)	1.06 (0.52–2.15)	1.04 (0.94–1.15)
Surfactant delivered with INSURE	8.4	7.2	0.97	0.98 (0.38–2.54)	0.93 (0.35–2.43)	0.51 (0.19–1.4)	1.16 (0.97–1.39)
Rapid FiO_2_ reduction documented post-administration	37.4	57.3	0.005^*^	2.21^*^ (1.26–3.88)	2.16^*^ (1.23–3.82)	1.29 (0.66–2.52)	1.1 (0.99–1.21)
Received early rescue surfactant	75	66.7	—	—	—	—	—
Repeat doses of Surfactant given if ongoing MV/raised FiO_2_	38.3	32.6	—	—	—	—	—
**Supportive Care**							
TPN Started on day one of life	74.7	84.4	0.001*	2.33* (1.43–3.79)	2.75^*^ (1.66–4.58)	1.4 (0.83–2.39)	0.74^*^ (0.65–0.84)
Minimal enteral feeding started on day one of life	32.4	37	0.24	1.25 (0.86–1.82)	1.18 (0.8–1.73)	0.86 (0.56–1.31)	1.22^*^ (1.12–1.32)

^*^Significant result, ^a^p-value from Chi-square test, ^b^OR from logistic regression model comparing cohort II with cohort I, ^c^OR from logistic regression model adjusted for level of unit and gestation at birth, comparing cohort II to cohort I. ^d^OR from logistic regression model comparing level 3 units with level 2 units, ^e^OR from logistic regression model showing odds for every unit increase in gestational age at birth.

OR = Odds Ratio, GA = Gestational Age, PPROM = preterm prelabour rupture of membranes, FiO_2_ = Fraction of inspired oxygen, CPAP = Continuous positive airway pressure support, TTV = Targeted Tidal Volume, PEEP = Positive End Expiratory Pressure, MV = Mechanical Ventilation, TPN = Total Parenteral Nutrition.

### Antenatal management

Rates of any antenatal steroid exposure in both cohorts were high, with no significant difference (92.4% in 2015 vs. 89.5% in 2018 p = 0.31; aOR 0.74 [95% CI 0.4, 1.4]). Likelihood of antenatal steroid exposure did not vary depending on GA or unit level. Timings of antenatal steroid administration were similar, with 70.7% receiving two doses >24 hours prior to delivery in 2015, compared to 70% in 2018 (p = 0.3). 11% in 2015 had a complete course <24 hours prior to delivery, compared to 11.7% in 2018 (p = 0.75).

### Delivery room stabilisation

A minority of infants in both cohorts received delayed cord clamping (DCC) (12% vs. 13%, p = 0.23; aOR 1.30 [0.75, 2.27]). The unit level did not have a significant effect on this practice. More mature infants were more likely to receive DCC (aOR 1.44 [1.23, 1.68]). Initiation of stabilisation of preterm infants is recommended with 21–30% fractional inspired oxygen (FiO_2_). There was a significant reduction in the proportion of infants managed in this way (76.4% vs 66.3%, p < 0.001, aOR 0.29 [0.17, 0.47]). This reduction was not affected by unit level; however more premature infants were less likely to be stabilised in an FiO_2_ below 30% (aOR 0.74 [0.67, 0.79]).

For infants born at <28 weeks’ gestation it is recommended that they be delivered into a plastic bag. Similar rates were seen in both cohorts (76.9% vs. 71.1%, p = 0.79, aOR 0.47 [0.08, 2.84]).

50.7% of infants were stabilised on Continuous Positive Airway Pressure (CPAP) in 2015, compared to 51.8% in 2018. 36.9% of infants required intubation in the delivery room in 2015, compared to 34.8% in 2018. No respiratory support was required in 1.3% of cases in 2015, and 6.5% in 2018. The likelihood of being stabilised with CPAP did not vary between cohorts (aOR 0.78 [0.47, 1.28]) but more premature infants were significantly more likely to be intubated (aOR 1.85 [1.65, 2.08]), and less likely to be stabilised on CPAP (aOR 0.54 [0.48, 0.61]).

### Non-invasive ventilation management

Among infants stabilised on non-invasive support, CPAP was the primary mode used in 92.1% in 2015 compared to 90.9% in 2018 (p = 0.31 aOR 0.53 [0.18, 1.60]). No significant variation was seen based on unit level or GA. A CPAP pressure of ≥6cmH_2_O is recommended; however, a significant decrease in the proportion of infants who are documented to have received a CPAP pressure ≥6cmH_2_O was seen (50.9% vs 37.8%, p = 0.01, aOR 0.58 [0.35, 0.96]). Infants in level three units (aOR 1.8 [1.02, 3.19]) and more premature infants (aOR 1.2 [1.04, 1.41]) were more likely to have received ≥6cmH_2_O.

### Mechanical ventilation strategies

A significant increase in use of Targeted Tidal Volume (TTV) mode was observed (49.1% vs 79.1%, p =<0.001, aOR 7.94 [3.75, 16.8]). No difference was seen based on unit level; however more premature infants were more likely to receive TTV (aOR 1.14 [1.01, 1.3]). There is no standard ventilator model in use across the Wales Neonatal Network, but all ventilators in clinical use across level 2 and 3 units contain a volume-targeted ventilation algorithm.

There was a significant increase in caffeine use between the cohorts (75.6% vs 81.9%, p = 0.03, aOR 2.49 [1.4, 4.6]) with more premature infants being more likely to receive caffeine (aOR 1.79 [1.45, 2.22]).

### Surfactant therapy

All infants who had surfactant replacement therapy received a natural surfactant preparation. Rates of infants receiving surfactant if requiring intubation for stabilisation in the delivery room remained consistently high (100% of 2015 cohort and 97.9% of 2018 cohort (p = 0.19)).

InSurE (Intubate Surfactant Extubate) is a recommended mode for surfactant delivery. Of all eligible infants receiving surfactant, there was no increase in InSurE between cohorts (8.4% vs 7.2%, p = 0.97). Less invasive surfactant administration (LISA) was practiced for 9 infants in the 2018 cohort.

A dose of 200 mg/kg of surfactant is recommended; however less than half of eligible infants in both cohorts received this dose (49.5% vs 45.7%, p = 0.26, aOR 1.38 [0.77, 2.49]). The rest received a lower dose per kilogram of birth weight. No difference was seen based on unit level or GA. Following surfactant administration, it is recommended that FiO_2_ should be reduced quickly. The documentation of this practice significantly improved (37.4% vs 57.3%, p = 0.005 aOR 2.16 [1.23, 3.82]).

### Supportive care

It is recommended that all preterm infants start parenteral nutrition (PN) and minimal enteral feeding on day one of life. PN use showed a significant increase (74.7% vs 84.4%, p = 0.001, aOR 2.75 [1.66, 4.58]), although more premature infants were more likely to receive PN (aOR 1.35 [1.19, 1.54]). Rates of minimal enteral feeding on day one remained similar between cohorts (32.4% vs 37%, p = 0.24, aOR 1.18 [0.8, 1.73]), and more mature infants were more likely to start feeds on day one of life (aOR 1.22 [1.13, 1.32]).

### Subgroup analyses

The results from the post-hoc subgroup analyses were broadly similar to the regression analyses. All results are presented in Supplementary Tables 2 and 3.

In the <28-week gestation subgroup, a significant decrease was seen in the number of infants stabilised in FiO_2_ 21–30% (72.1 vs 22.2% p =< 0.001). A significant decrease was also seen in DCC (9.8% vs 0% p = 0.04). A significant increase in the number receiving TTV (67.5 vs 92.3% p = 0.006) was observed. In the ≥28-weeks subgroup a significant decrease was seen in stabilisation in FiO_2_ 21–30% (87.6 vs 75.9% p = 0.004). Significant improvements were observed in the use of TTV (47 vs 88.6% p =< 0.0001), caffeine (79.4 vs 88.1% p = 0.02), FiO_2_ reduction post-surfactant administration (44.2 vs 70.4% p = 0.003), and TPN starting on day 1 of life (72.1 vs 86% p = 0.001). However, a significant decrease in the use of CPAP pressures ≥ 6cmH_2_O was also seen (53.6 vs 37.9% p = 0.014).

In the level 2 subgroups analysis significant differences were seen in surfactant dosing of 200 mg/kg (45.5 vs 76.2% p = 0.04), FiO_2_ reduction post-surfactant administration (31.8 vs 71.4% p = 0.005) and TPN started on day one of life (66.1 vs 86.5% p = 0.006). In the level 3 subgroup analysis, significant decreases were seen in the proportion of infants stabilised in FiO_2_ 21–30% (83 vs 4.1% p =< 0.0001) and the use of CPAP pressures of ≥ 6cmH_2_O (60.7 vs 41.9% p = 0.011). Significant improvements were seen in the use of TTV (54.9 vs 93.3% p =< 0.0001), caffeine prescribing (82.6 vs 90.2% p = 0.04) and TPN use on the first day of life (79.9 vs 88.9% p = 0.018).

## Discussion

Early management of RDS can have a significant impact on later morbidity, especially on the development of CLD^13^. An extensive evidence base has grown over the past two decades on optimal management for these vulnerable infants^8,9,12^. We present for the first time the assessment of RDS management across several Welsh neonatal units before and after the implementation of a national guideline. To our knowledge, this is the first time RDS management has been reported in a relatively large cohort over time against the same recommendations in the UK.

There were several notable improvements in management, the most significant of which are use of TTV-mode, FiO2 management post-surfactant administration, increased caffeine prescribing, and increasing use of parenteral nutrition on day one of life. However, there are two areas which appear to have deteriorated: stabilising infants in the delivery room with an FiO2 21–30% and use of CPAP pressures of ≥ 6cmH2O. Both of these practices showed some variation depending on the unit level and GA, with the tertiary units being more likely to use higher CPAP pressures, but less mature infants being more likely to be stabilised with a higher inspired oxygen fraction. Owing to the data collection methodology, this could be secondary to documentation; however, it is important to emphasise the importance of not exposing premature infants to unnecessarily high FiO2 during stabilisation.

Immediate management of an infant being born prematurely is a multi-disciplinary process in the delivery room. Despite evidence supporting the efficacy of DCC in premature infants^14^, rates remained low in both cohorts, with no significant improvement. However, there was significant evidence of a move towards DCC for more mature infants.

Additionally, minimal enteral feeding commencing on day one of life remains low and unimproved. This may be secondary to clinical concerns with the infant but may also be due to a lack of maternal expressed breast milk. This often requires support from the midwifery team in the first hours of life. This highlights the need for robust training and complementary guidance between departments to ensure optimal practice. The recently published SIFT trial^15^ has demonstrated the safety of establishing full enteral feeding early in preterm and very-low-birthweight infants, and this evidence will hopefully aid clinician’s confidence in initiating early enteral feeding in practice.

We observed variation in management in several areas based upon GA. More mature infants were more likely to be stabilised on non-invasive respiratory support. This may be due to the experience of those in attendance and general confidence within unit culture in managing more vulnerable infants with non-invasive ventilation. Extremely preterm infants born in level two units may need intubation before transfer to a tertiary unit for further care. However, no variation in intubation was observed based on unit of delivery, although only a small proportion of these infants were delivered in level two units. Further evidence on LISA in extreme preterm infants^16^ may improve the success of non-invasive respiratory support in this population. In addition, several local quality improvement projects are ongoing in many of the neonatal units in Wales, and we hope to see improved practice in the next round of data collection.

Our findings are in keeping with the limited amount of published data on RDS management against consensus guidance. A UK-based survey from 2018 found a significant number of units reluctant to use CPAP as the primary ventilation mode for extremely premature infants, but TTV-mode use was increasingly popular for mechanically ventilated infants^17^. Single- centre retrospective audits have been published on adherence to aspects of previous consensus guidelines. Retrospective audits have found variable use of prophylactic surfactant with less use in more mature infants^18^, and good adherence on early management in a cohort of twenty infants <28 weeks’ gestation^19^. Both audits examined much more limited aspects of older European Consensus guidelines highlighting the need for robust training and education, but none published follow-up data to document changes.

Our study has several strengths. By capturing prospective data across multiple sites during the same time points, we have achieved a highly representative, contemporaneous impression of current practice across two epochs. This is the first report we are aware of describing changes of practice in RDS management within a defined population over time in the UK, which demonstrates the impact of a unified national guideline on RDS management. Our data collection was restricted mainly to areas of practice supported by Grade A evidence, and we used robust statistical methods to analyse reliable data. We believe this framework can be used in any neonatal network in the UK to document quality improvement in the management of RDS in preterm infants.

There are several limitations of our work. As with any multi-centre audit project there were missing data for variables, and this varied by cohort and data item (Supplementary Figs. 2 and 3). The effect of incomplete participation of level-two units was partly mitigated as the majority of preterm infants were delivered in tertiary units. However, some Welsh preterm infants born in non-participating units did not have their management audited, and there may be more variation in practice than appreciated. It was difficult to determine the number of eligible infants (denominator) for some interventions through the data collection process, although the majority of these were based on grade B and C evidence. We also did not collect data on other relevant clinical indicators like admission temperature, which may have an effect on the severity of RDS; this will be added in future rounds of data collection. Finally, since our study examined practice against an established guideline, data on the outcome of these infants were not collected; this remains an objective in future rounds of data-collection.

Our analysis demonstrates that certain desirable Grade A evidence-based interventions are still yet to come into clinical practice. The 2013 edition of the European consensus guideline recommended a change in practice towards stabilising preterm infants on non-invasive respiratory support at delivery rather than elective intubation^9^. Additionally, it also recommended delayed cord clamping of at least sixty-seconds is practised where possible. Our data demonstrates that intubation rates at delivery have remained unchanged despite introduction of the Wales guideline, and the number of infants receiving delayed cord clamping remains low. There are quality improvement and training initiatives targeting these practices currently underway in Welsh neonatal units, and this will form a major focus in the next cycle of data collection in this quality improvement project.

In conclusion, this study highlights the successes and challenges with improving management and reducing the variation in practice of RDS in Wales. Interestingly, our data suggests relatively limited variation between level two and three units. Some important areas of practice have shown substantial improvement, but there remain areas of practice that are not in keeping with current best evidence, most importantly the low rates of extremely preterm infants being stabilised on non-invasive respiratory support. Unified, national guidelines are a potentially powerful tool to effect change and reduce variation in practice. They are feasible to implement and can be established in other neonatal networks and nations of the UK. Moving forward, an update to the European Consensus Guideline has recently been published^13^ and, following review, the Wales Neonatal Network guideline will be updated to reflect any substantial changes, followed by dissemination of the evidence and education of staff. Re- evaluations of RDS management in Wales are planned to continue on a three-yearly cycle, with the next being undertaken in 2021. We aim to monitor progress and highlight areas requiring further education, cross-departmental working and training to optimise our RDS management.

## Supplementary information

Supplementary Information.

## Data Availability

All data relevant to the study is included in the manuscript and Supplementary Information.
