# Supplementary material for: Management of Respiratory Distress Syndrome in Preterm Infants In Wales: A Full Audit Cycle of a Quality Improvement Project
Source: Sci Rep. 2020 Feb 26;10:3536. doi: 10.1038/s41598-020-60091-6 (PMC7044423; doi:10.1038/s41598-020-60091-6)
Supplement: Supplementary file 1 — Supplementary Information. [file 41598_2020_60091_MOESM1_ESM.docx]

**Management of Respiratory Distress Syndrome in Preterm Infants In Wales: A Full Audit Cycle of a Quality Improvement Project**

**Christopher Course, Mallinath Chakraborty**

**Supplementary Information**

**Supplementary Materials:**

**Supplementary Figure 1: Gestational spread of both cohorts**

**
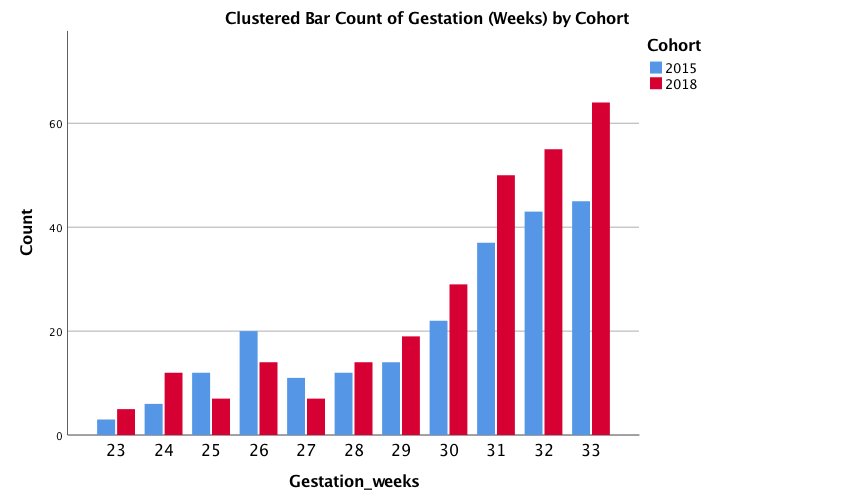
**

**Supplementary Figure 2: Flowchart detailing overall management of the 2015 cohort**

**
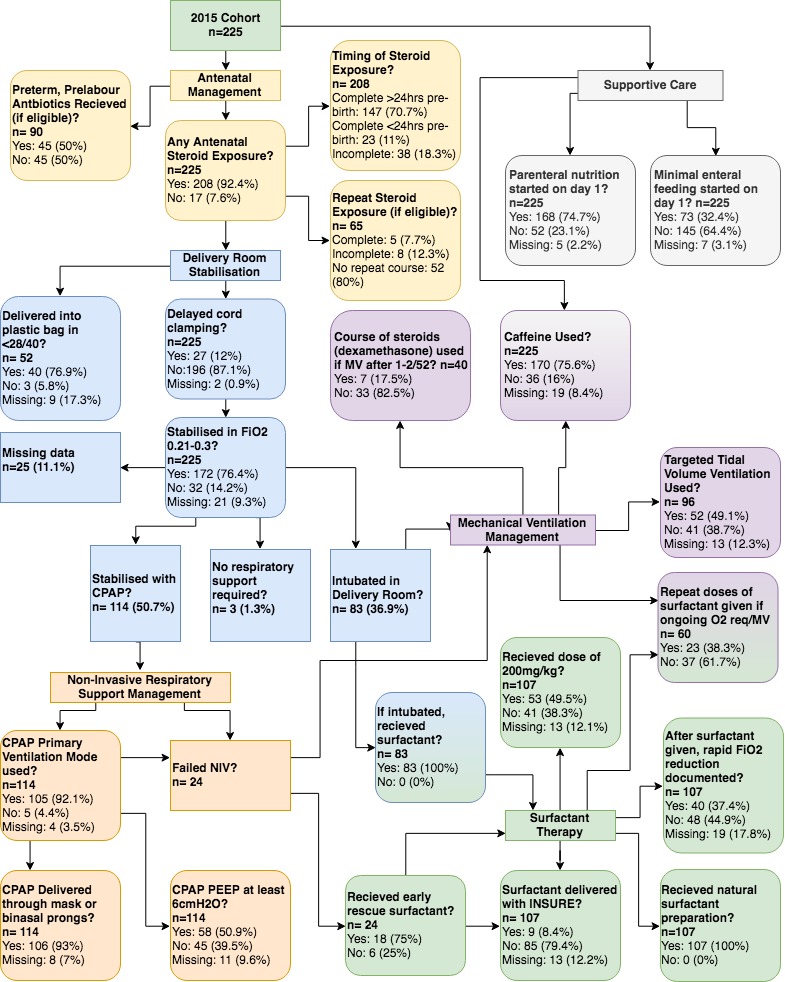
**

**Supplementary Figure 3: Flowchart detailing overall management of the 2018 cohort**

**
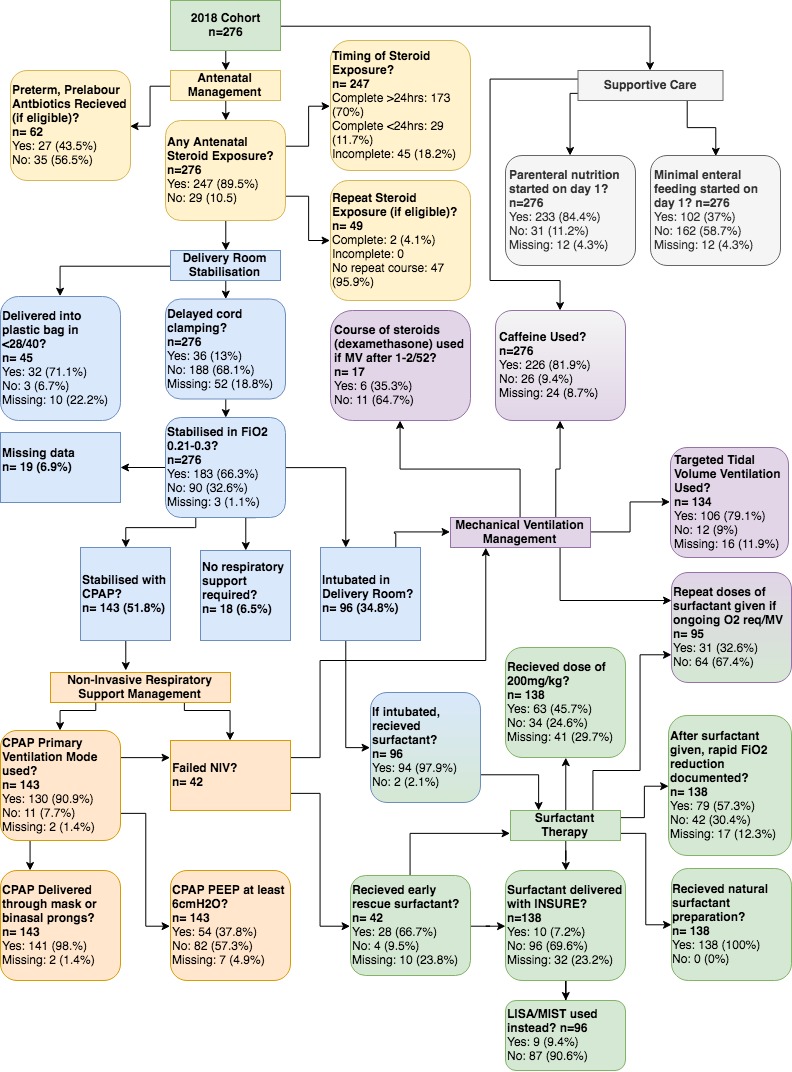
**

| **Demographics** | **Data** |
| --- | --- |
| Hospital of Delivery |  |
| Hospital Number |  |
| Gestation at Birth |  |
| Birth Weight (grams) |  |
| Transferred out to another unit? If so, which unit was baby transferred to? |  |
| **Prenatal Care: Mother** |  |
| Received a course of prenatal steroids if gestation between 23 and 34 weeks. * |  |
| Received a second course of steroids if first course administered more than 2-3 weeks before delivery. # |  |
| Received antibiotics if preterm, pre-labour rupture of membranes. |  |
| **Delivery Room Stabilisation $** |  |
| Cord clamping delayed for at least 60 seconds (if possible). |  |
| Stabilisation initiated in 21-30% oxygen. |  |
| Spontaneously breathing baby stabilised with PEEP, and CPAP used as first ventilation support on admission to unit. |  |
| If required intubation, received surfactant. |  |
| If <28 weeks gestation, delivered into a plastic bag. |  |
| **Surfactant Therapy $** |  |
| Received a natural surfactant preparation (if needed). |  |
| Received early rescue surfactant if: • <26 weeks and FiO2 >30% • >26 weeks and FiO2 >40% |  |
| Received a rescue dose of 200mg/kg surfactant |  |
| INSURE technique used for rescue surfactant administration. |  |
| Repeat doses of surfactant given in ongoing evidence of RDS, e.g. O2 requirement/need for MV |  |
| After surfactant given, rapid reduction in administered FiO2 documented. |  |
| **Non-Invasive Respiratory Support $** |  |
| CPAP first-line respiratory support used for RDS, if not intubated. |  |
| CPAP delivered through mask or bi-nasal prongs |  |
| CPAP pressure of at least 6 cm of water applied |  |
| Mechanical Ventilation Strategies |  |
| If baby intubated, documented reason why: • Elective/Prophylactic • Failing NIV • Sepsis  • For transfer to another unit • Any reason other than RDS |  |
| Targeted tidal volume ventilation used. $ - If not used, what is documented reason why?  (e.g. high leak, other lung pathology etc.) |  |
| Caffeine used in baby if; apnoea, or, facilitate weaning from MV. $ |  |
| Tapering course of steroids (dexamethasone) used if remain on MV after 1-2 weeks. $ |  |
| Supportive Care $ |  |
| Parenteral nutrition started on day 1 of life. |  |
| Minimal enteral feeding/trophic feeds started on day 1 of life. |  |

**Supplementary Table 1:** Data Collection Proforma. (* 1=complete course ≥ 24 hours before delivery, 2=complete course but ≤ 24 hours before delivery, 3=incomplete course, 4=no steroid exposure; # 1=complete second course of steroids, 2=incomplete second course of steroids, 3=no second course of steroids; $ 1=yes, 2=no)

| **< 28 weeks Gestational Age at Delivery** | | | | | | |
| --- | --- | --- | --- | --- | --- | --- |
|  | | **2015 Cohort n(%)** | | **2018 Cohort n(%)** | | **p-value** |
| Number of infants | | 52 | | 45 | | x |
| No steroid exposure | | 5(9.6) | | 2(4.4) | | 0.127 |
| Any steroid exposure | | 47(90.3) | | 43(95.5) | | 0.326 |
| Delayed Cord Clamping | | 5(9.8) | | 0(0) | | 0.04* |
| Stabilised in FiO2 21-30 | | 31(72.1) | | 10(22.2) | | <0.001* |
| Stabilised with CPAP | | 5(10.4) | | 6(14.6) | | 0.55 |
| Stabilised with intubation | | 43(89.6) | | 35(85.4) | | 0.55 |
| CPAP Primary NIV mode used on admission | | 4(80) | | 4(66.7) | | 0.62 |
| PEEP ≥6cmH2O used | | 6(75) | | 4(100) | | 0.27 |
| TTV Used | | 27(67.5) | | 36(92.3) | | 0.006* |
| Caffeine used to wean MV/prevent apnoea | | 47(92.2) | | 41(97.6) | | 0.25 |
| Received 200mg/kg surfactant | | 21(55.3) | | 19(54.3) | | 0.93 |
| Surfactant delivered with InSurE | | 1(2.7) | | 2(5.6) | | 0.54 |
| Rapid FiO2 reduction post surfactant documented | | 17(48.6) | | 22(55%) | | 0.58 |
| TPN Started Day 1 of life | | 44(91.7) | | 42(100) | | 0.06 |
| Minimal enteral feeding started day 1 of life | | 13(27.7) | | 6(14.3) | | 0.12 |
| **28 - 33+6 weeks Gestational Age at Delivery** | | | | | | |
|  | **2015 Cohort n(%)** | | **2018 Cohort n(%)** | | **p-value** | |
| Number of infants | 173 | | 231 | | x | |
| No steroid exposure | 12(6.9) | | 26(11.3) | | 0.34 | |
| Any steroid exposure | 161(93.1) | | 205(88.7) | | 0.14 | |
| Delayed Cord Clamping | 22(12.8) | | 36(19.5) | | 0.09 | |
| Stabilised in FiO2 21-30 | 141(87.6) | | 173(75.9) | | 0.004* | |
| Stabilised with CPAP | 109(74.1) | | 137(69.2) | | 0.31 | |
| Stabilised with intubation | 38(25.9) | | 61(30.8) | | 0.31 | |
| CPAP Primary NIV mode used on admission | 101(96.2) | | 126(93.3) | | 0.33 | |
| PEEP ≥6cmH2O used | 60(53.6) | | 50(37.9) | | 0.014* | |
| TTV Used | 25(47) | | 70(88.6) | | <0.0001* | |
| Caffeine used to wean MV/prevent apnoea | 123(79.4) | | 185(88.1) | | 0.02* | |
| Received 200mg/kg surfactant | 32(58.2) | | 44(71.0) | | 0.15 | |
| Surfactant delivered with InSurE | 8(14.0) | | 8(11.4) | | 0.66 | |
| Rapid FiO2 reduction post surfactant documented | 23(44.2) | | 57(70.4) | | 0.003* | |
| TPN Started Day 1 of life | 124(72.1) | | 191(86) | | 0.001* | |
| Minimal enteral feeding started day 1 of life | 60(35) | | 96(43) | | 0.10 | |

**Supplementary Table 2:** Subgroup analyses based on gestation at birth (* = p < 0.05)

| **Delivered in Level 2 Unit** | | | |
| --- | --- | --- | --- |
|  | **2015 Cohort n (%)** | **2018 Cohort n (%)** | **p-value** |
| Number of infants | 59 | 77 | X |
| No steroid exposure | 3(5.1) | 8(10.4) | 0.60 |
| Any steroid exposure | 56(94.9) | 69(89.6) | 0.26 |
| Delayed Cord Clamping | 5(8.5) | 10(15.9) | 0.21 |
| Stabilised in FiO2 21-30 | 45(88.2) | 56(74.7) | 0.06 |
| Stabilised with CPAP | 28(54.6) | 46(71.9) | 0.06 |
| Stabilised with intubation | 23(45.1) | 18(28.1) | 0.06 |
| CPAP Primary NIV mode used on admission | 27(100) | 42(93.3) | 0.17 |
| PEEP ≥6cmH2O used | 12(38.7) | 15(34.9) | 0.74 |
| TTV Used | 13(59.1) | 23(79.3) | 0.12 |
| Caffeine used to wean MV/prevent apnoea | 42(82.4) | 60(88.2) | 0.36 |
| Received 200mg/kg surfactant | 10(45.5) | 16(76.2) | 0.04* |
| Surfactant delivered with InSurE | 3(13.6) | 4(17.4) | 0.73 |
| Rapid FiO2 reduction post surfactant documented | 7(31.8) | 20(71.4) | 0.005* |
| TPN Started Day 1 of life | 37(66.1) | 64(86.5) | 0.006* |
| Minimal enteral feeding started day 1 of life | 21(36.8) | 34(45.3) | 0.33 |
| **Delivered in Level 3 Unit** | | | |
|  | **2015 Cohort n (%)** | **2018 Cohort n (%)** | **p-value** |
| Number of infants | 166 | 199 | - |
| No steroid exposure | 14(8.4) | 20(10) | 0.79 |
| Any steroid exposure | 152(91.6) | 179(89.9) | 0.60 |
| Delayed Cord Clamping | 22(13.4) | 26(16.1) | 0.49 |
| Stabilised in FiO2 21-30 | 127(83.0) | 127(64.1) | <0.0001* |
| Stabilised with CPAP | 86(59.7) | 97(55.4) | 0.44 |
| Stabilised with intubation | 58(40.3) | 78(44.6) | 0.44 |
| CPAP Primary NIV mode used on admission | 78(94.0) | 88(91.7) | 0.55 |
| PEEP ≥6cmH2O used | 54(60.7) | 39(41.9) | 0.011* |
| TTV Used | 39(54.9) | 83(93.3) | <0.0001* |
| Caffeine used to wean MV/prevent apnoea | 128(82.6) | 166(90.2) | 0.04* |
| Received 200mg/kg surfactant | 43(60.6) | 47(61.8) | 0.87 |
| Surfactant delivered with InSurE | 6(8.3) | 6(7.2) | 0.80 |
| Rapid FiO2 reduction post surfactant documented | 33(50.8) | 59(63.4) | 0.11 |
| TPN Started Day 1 of life | 131(79.9) | 169(88.9) | 0.018* |
| Minimal enteral feeding started day 1 of life | 52(32.3) | 68(36.0) | 0.47 |

**Supplementary Table 3:** Subgroup analysis based on level of unit at birth (* = p < 0.05)
